# [Retracted] Puerarin promotes DUSP1 expression by regulating miR-133a-3p in breast cancer

**DOI:** 10.3892/mmr.2026.13945

**Published:** 2026-06-23

**Authors:** Zhifeng Li, Weiwei Xu, Xiaoyan Ren, Jinhua Xu, Jianxin Chen

Mol Med Rep 19: 205–212, 2019; DOI: 10.3892/mmr.2018.9682

Following the publication of the above paper, it was drawn to the Editor's attention by a concerned reader that the control β-actin blots shown in Fig. 3A on p. 209 had either appeared previously, or were subsequently to appear, in three other articles in different journals written by different authors at different research institutes. In addition, an independent analysis of the data in this paper undertaken by the Editorial Office revealed that the western blots for the Bax protein in Fig. 3A, the control β-actin blots in Fig. 4A and all the western blot data shown in Fig. 5A and C had also been included in five other papers written by different authors/research groups, the majority of which had already been published or were under consideration for publication when the above article was received at *Molecular Medicine Reports*.

Owing to the fact that the contentious data in the above article had already been published prior to its submission to *Molecular Medicine Reports*, the Editor has decided that this paper should be retracted from the Journal. The authors were asked for an explanation to account for these concerns, but the Editorial Office did not receive a satisfactory reply. The Editor apologizes to the readership for any inconvenience caused.

